# Nonsurgical periodontal treatment reduced aortic inflammation in ApoE^−/−^ mice with periodontitis

**DOI:** 10.1186/s40064-016-2637-z

**Published:** 2016-06-30

**Authors:** Di Cui, Houxuan Li, Lang Lei, Changxing Chen, Fuhua Yan

**Affiliations:** Nanjing Stomatological Hospital, Medical School of Nanjing University, 30 Zhong Yang Road, Nanjing, 210008 Jiangsu People’s Republic of China

**Keywords:** Atherosclerosis, Lipid, Periodontitis, SRP

## Abstract

**Background:**

Although the correlation between periodontal infection and atherosclerotic lesions has been well recognized, whether and how the nonsurgical periodontal treatment (NSPT) can improve the vascular inflammation has not been investigated clearly.

**Methods:**

Thirty-two apolipoprotein E^−/−^ (apoE^−/−^) mice were randomly divided into four groups: (1) Con group: no treatment, blank control group; (2) Lig group: ligature-induced-periodontitis group; (3) Lig-N group: ligatures were removed on the 7th day; (4) Lig-SRP group: ligatures were removed on the 7th day, and scaling and root planing (SRP) were performed on the 9th day. All the animals were euthanized on the 30th day. Alveolar bone loss (ABL) was assessed under microcomputed tomography. Systemic inflammatory status and lipid contents in the plasma were detected. Expression of several surrogate markers for vascular inflammation was evaluated by immunohistology and quantitative real time PCR.

**Results:**

NSPT reduced ABL, improved lipid profile, and inhibited systemic inflammation with reduced plasma interleukin-6 (IL-6) level in apoE^−/−^ mice; in addition, reduced inflammation in arterial wall was observed in NSPT treated mice, showing less vascular cell adhesion molecule-1 expression and less macrophage adhesion; furthermore, NSPT improved elastic fiber fragmentation disorder in the aortic wall, thus preserved elasticity of aortic artery.

**Conclusion:**

Ligature-induced periodontitis can lead to inflammatory response in the vascular wall and NSPT has beneficial effect on the early stage of atherosclerosis process in the articular wall by reducing systemic inflammation and improving lipid profile.

## Background

Atherosclerosis, the major pathology underlying cardiovascular disorder, is considered as an inflammatory disease, characterized by intense immunological activity in the arteries (Ross 1999). One particular characteristic of atherosclerosis is the increased expression of various inflammatory mediators such as vascular cell adhesion molecule-1 (VCAM-1) (Mackesy and Goalstone 2011) and matrix metallopeptidase-9 (MMP-9), which is critical for the development of arterial lesions by regulating smooth muscle cell migration and matrix degradation (Cho 2002), in arterial lesions.

Inflammation that extends deep into the periodontal tissues and causes loss of supporting connective tissue and alveolar bone is known as periodontitis (Pihlstrom et al. 2005). It is an inflammatory disease initiated by polymicrobial dysbiosis (Hajishengallis and Lambris 2012).

A relationship between periodontal disease and atherosclerosis has been recognized in epidemiological studies (Lockhart et al. 2012). Periodontitis can elicit endothelial dysfunction, oxidative stress, and lipid accumulation in the aorta (Abe and Hajishengallis 2013; Miyajima et al. 2014; Brito et al. 2013); in addition, nonsurgical periodontal therapy (NSPT), scaling and root planing (SRP) primarily, improved lipid status and decreased systemic inflammatory response (Tawfig 2015; D’Aiuto et al. 2006; Hada et al. 2015). For example, SRP decreased serum tumor necrosis factor-α (TNF-α), interleukin-6 (IL-6) and C-reaction protein (CRP) levels in chronic periodontitis subjects with stable coronary heart disease (Tawfig 2015). Intensive periodontal therapy (SRP followed by the adjunctive use of a locally delivered antimicrobial) reduced systemic inflammatory markers and systolic blood pressure, and improved lipid profiles with subsequent changes in cardiovascular risk (D’Aiuto et al. 2006).

Although it has been demonstrated that periodontal treatment reduced lipid profiles and systemic inflammatory markers in epidemiological studies, whether and how NSPT can improve atherosclerotic status in the vascular wall has never been investigated. In the current study, we demonstrated for the first time that NSPT decreased inflammatory response in aortic arterial wall with reduced elastic fiber fragmentation, matrix degradation and VCAM-1 expression.

## Results

### NSPT reduced bacteria accumulation and periodontal inflammation

**Fig. 1:**
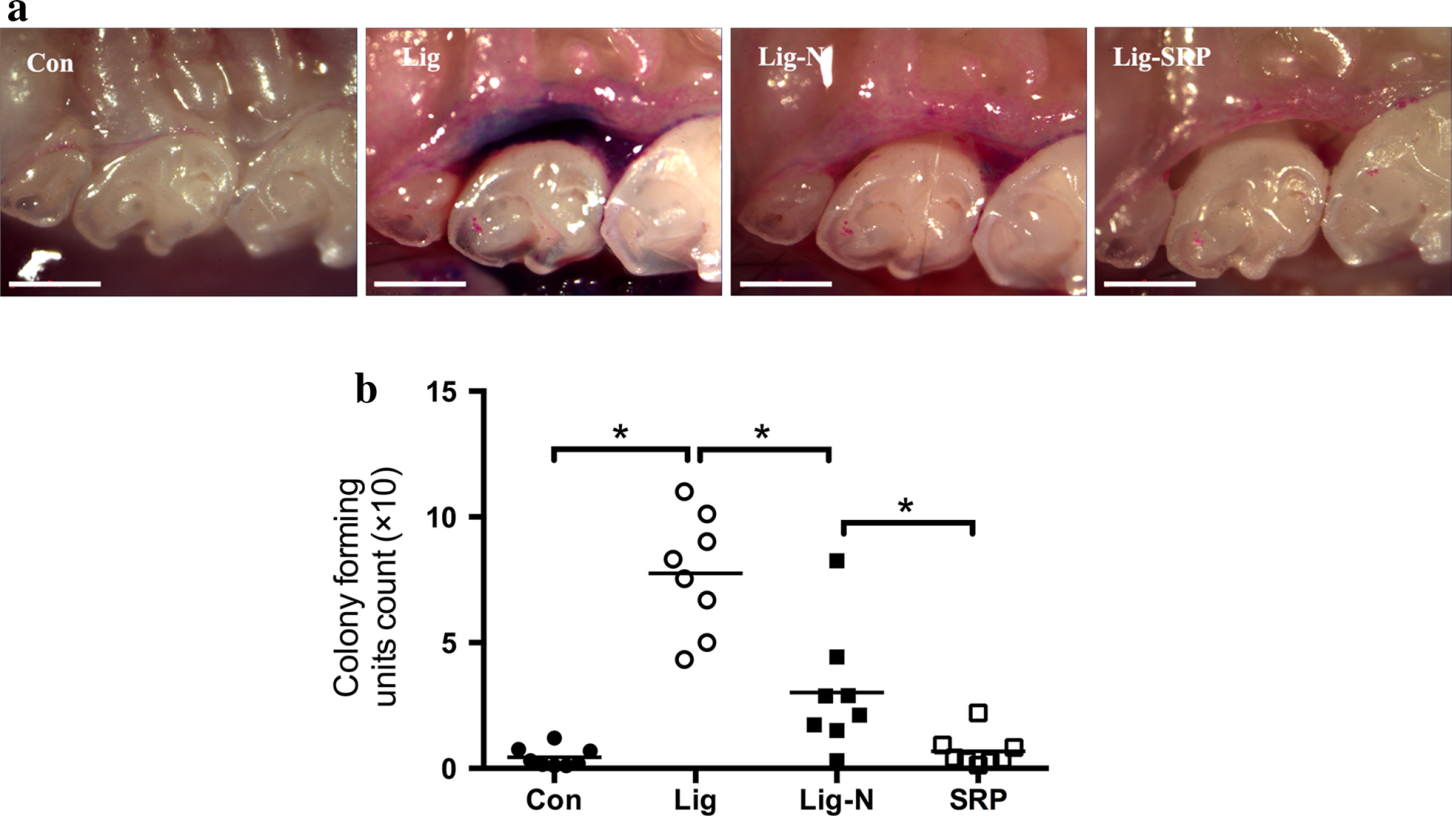
Determination of bacterial accumulation. **a** Dental plaque-disclosing photographs of the cervical portion of the M2 in mice. Older plaque is revealed in *blue*, newer plaque in *pink*. *Scale bar* 1 mm. **b** Quantification of anaerobic bacteria. Bacteria were extracted from recovered sutures and sterile paper points used to sample microbiota on the tooth surface and in the gingival crevicular (or periodontal pocket). Serial dilutions of bacterial suspensions were plated onto blood agar plates for anaerobic growth and CFU enumeration. Each *symbol* represents an individual mouse; *small horizontal lines* indicate the mean. *p < 0.05

Few supragingival plaques were observed in the control mice, while significant plaque accumulation was observed 7 d after ligature placement (p < 0.0001). Quantity of the plaques was reduced after removal of ligatures (p < 0.01), and SRP further reduced the biofilm quantity (p < 0.05) (Fig. 1).

**Fig. 2:**
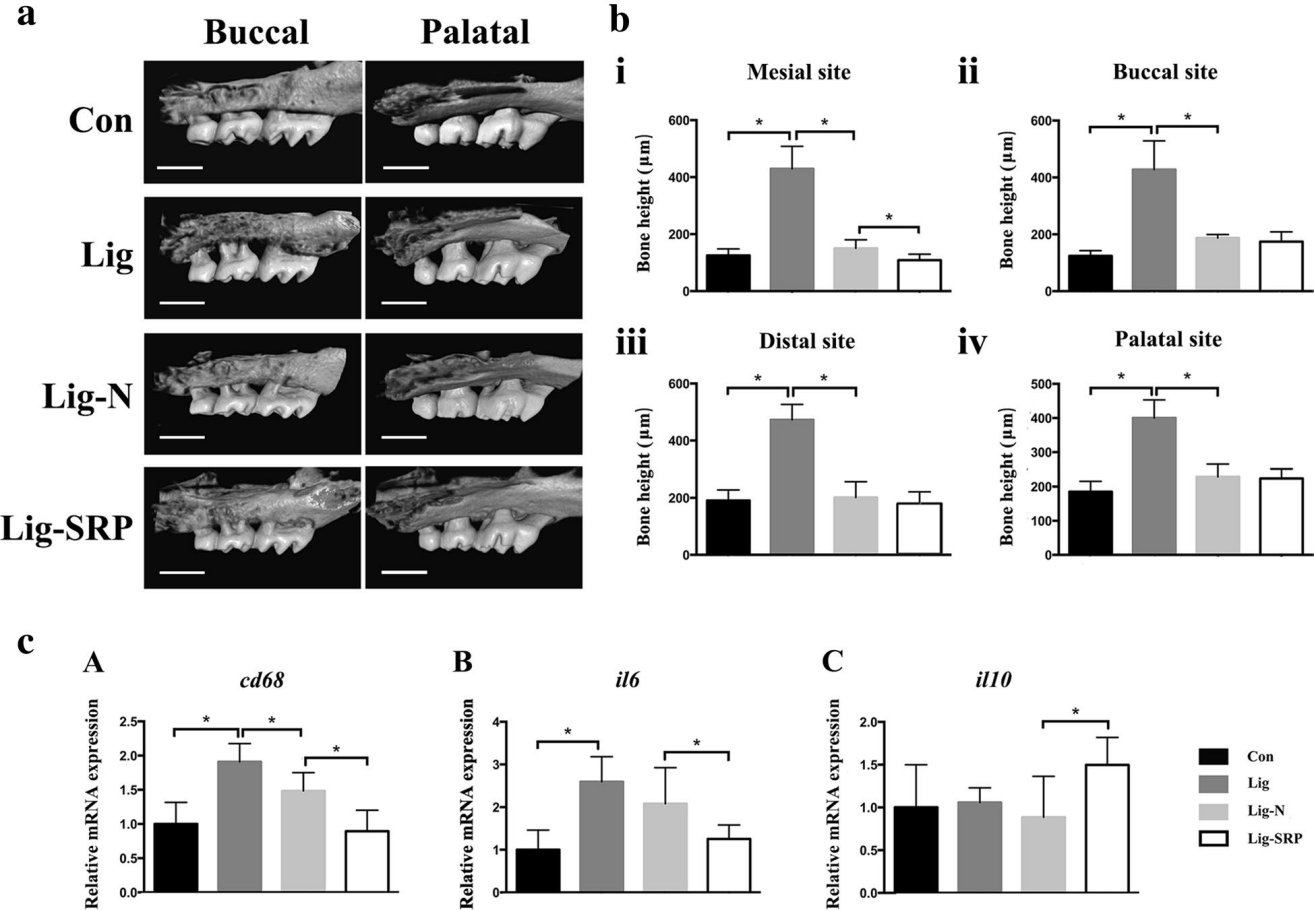
Identification of alveolar bone loss and periodontal inflammation. **a** Micro-CT images of maxillae. *Scale bar* 1 mm. **b** Results of bone height measurement. Distances from cement-enamel junction to the alveolar bone crest of the second molar at the mesial-bifurcation (**i**), buccal-bifurcation (**ii**), distal-bifurcation (**iii**) and palatal-groove (**iv**) site were evaluated. All results are expressed as the mean ± SD. *p < 0.05. **c** Relative gene expression levels in the periodontal tissue. The relative quantity of GAPDH mRNA was used to normalize the relative quantity of experimental mRNA: CD68 (**A**), IL-6 (**B**) and IL-10 (**C**). All results were expressed as the mean ± SD. *p < 0.05

Bone height was measured at four sites (Fig. 2). Significant alveolar bone loss was observed in the Lig group at all sites, and bone height at all the four sites was significantly decreased in the Lig-N group and the SRP group when compared with the Lig group (p < 0.01). At the mesial-bifurcation site (Fig. 2b i), no significant change was noted in Lig-N group or the SRP group, compared with the Con group. At the buccal-bifurcation site (Fig. 2b ii), when compared with the Con group, bone height was significantly increased in the other three groups (p < 0.05). At the distal-bifurcation site (Fig. 2b iii), no significant change was noted in Lig-N group or the SRP group, compared with the Con group. At the palate-groove site (Fig. 2b iv), when compared with the Con group, bone height was significantly larger in the other three groups (p < 0.01).

We further analyzed periodontal related molecules in periodontal tissues (Fig. 2c). CD68 is restricted to cells of myeloid lineage, especially monocytes/macrophages (Hume 2011). Expression levels of CD68 increased to 1.9 folds in the Lig group (p < 0.001) (Fig. 2c A). After the removal of ligatures with or without SRP, this data dropped to 0.9 and 1.5 folds separately (p < 0.05). Expression levels of IL-6 increased to 2.6 folds in the Lig group (p < 0.0001) (Fig. 2c B). After the removal of ligatures with or without SRP, this data dropped to 1.3 (p < 0.05) and 2.1 folds (p > 0.05) separately. In addition, the procedure of SRP resulted in the significant 1.5-fold-increase of IL-10 expression level (p < 0.05) (Fig. 2c C).

### Blood lipid response

**Fig. 3:**
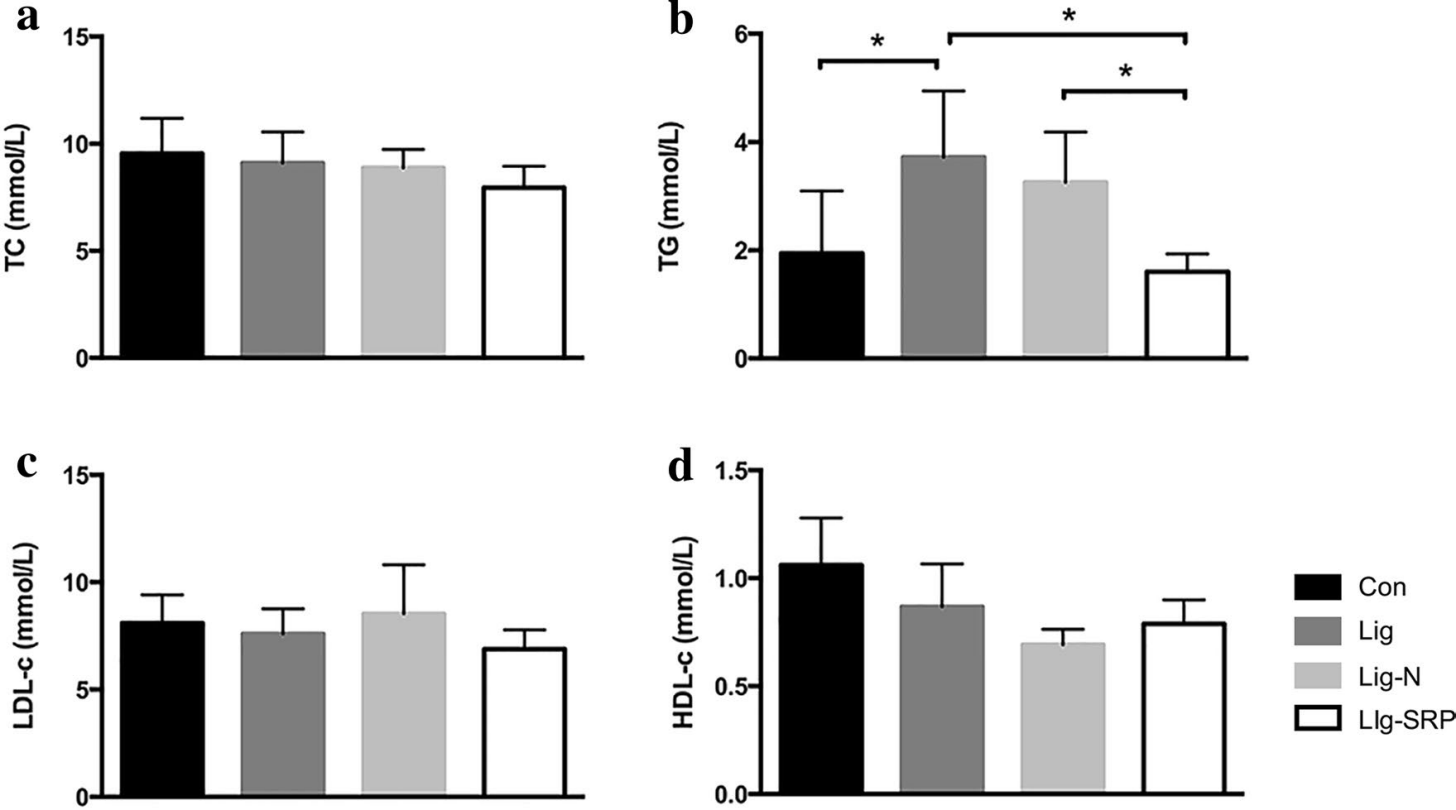
Plasma lipid profile. **a** Level of cholesterol (TC); **b** level of triglycerides (TG); **c** level of low-density lipoprotein cholesterol (LDL-c); and **d** level of HDL-c were detected. All results are expressed as the mean ± SD. *p < 0.05

Levels of total cholesterol (TC) in the Lig-N group and the Lig-SRP group were lower than the Lig group, but no significant difference was noted (Fig. 3a). Ligature-induced periodontitis significantly increased the level of triglycerides (TG) in apoE^−/−^ mice (p < 0.05), whereas removing the causal ligature decreased TG level in periodontitis mice; and SRP further improved TG level in the plasma of apoE^−/−^ mice (p < 0.05) (Fig. 3b). No significant difference in levels of low-density lipoprotein cholesterol (LDL-c) was observed among the four groups (Fig. 3c). Compared with the Con group, levels of HDL-c decreased in the Lig group, and a further reduction was observed in the Lig-N group, but no significant difference was noted (Fig. 3d). After the treatment of SRP, HDL-c levels increased slightly.

### System inflammatory response

**Fig. 4:**
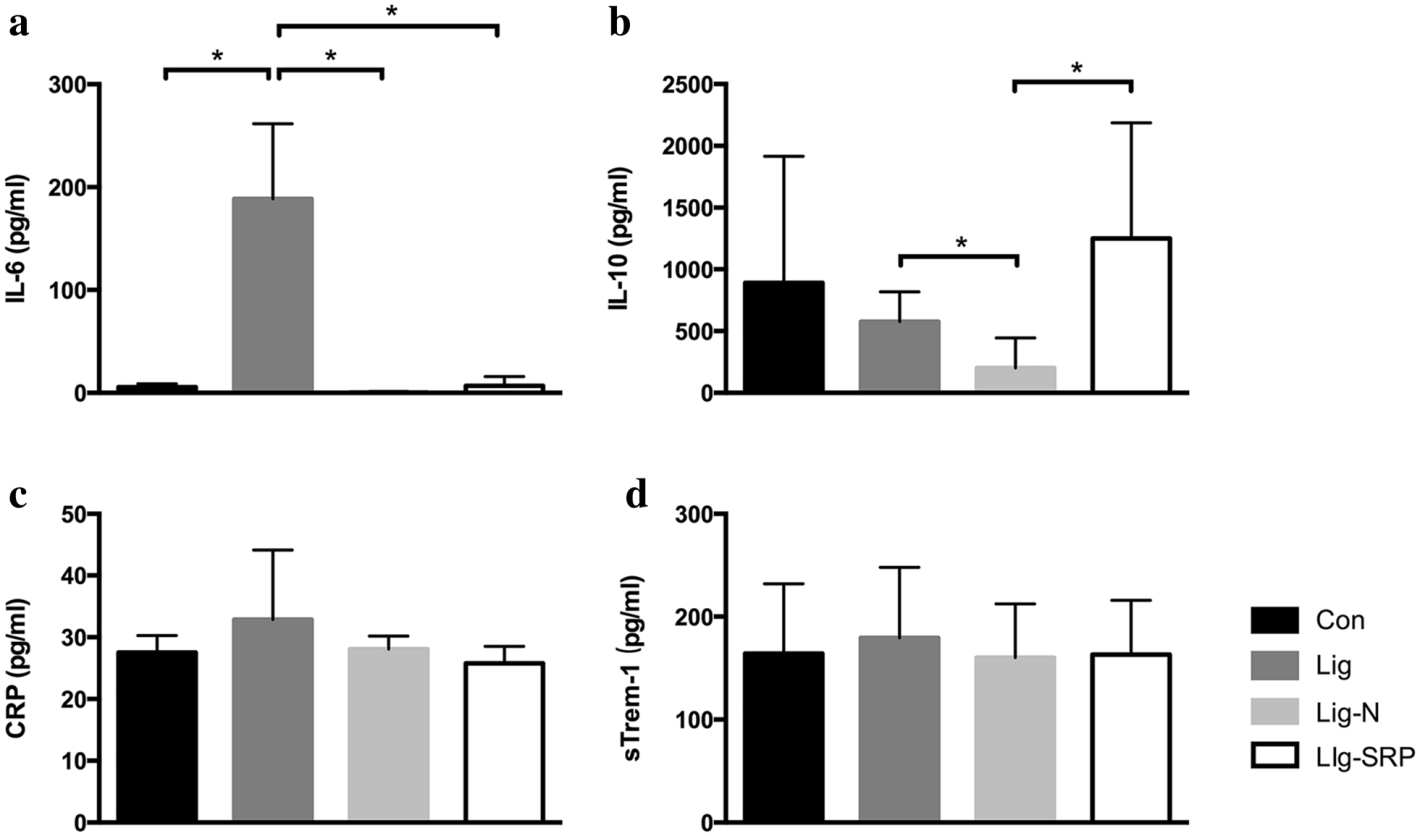
Inflammatory mediators in plasma. Levels of IL-6 (**a**), IL-10 (**b**); C-reactive protein (CRP) (**c**) and soluble triggering receptors expressed on myeloid cells-1 (sTrem-1) (**d**) in plasma was detected by ELISA. All results are expressed as the mean ± SD. *p < 0.05

While the pro-inflammatory IL-6 was undetectable in the Con group, the Lig-N group and the Lig-SRP group, the level of IL-6 was significantly increased in the Lig group (Fig. 4a). Regarding the anti-inflammatory IL-10 level, no difference was observed between the control and periodontitis group; in contrast, the level of IL-10 decreased significantly in the Lig-N group and increased significantly in the Lig-SRP group, when compared with the Lig group (Fig. 4b). No significant difference in the level of CRP and soluble triggering receptors expressed on myeloid cells-1 (TREM-1), a potential marker of infection, was observed among the four groups (Fig. 4c, d).

### Elastic fiber fragmentation

**Fig. 5:**
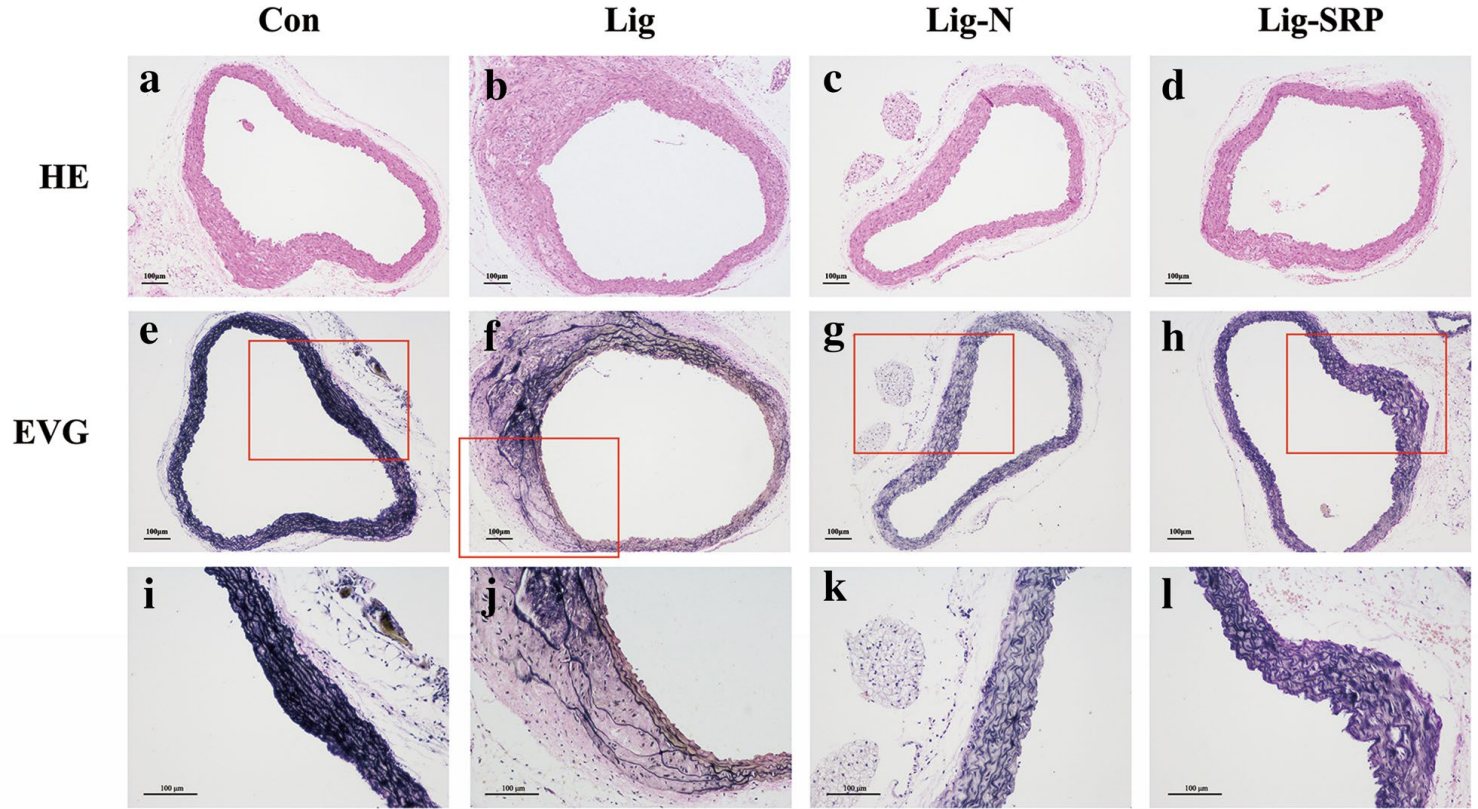
Histological changes and elastic fiber fragmentation in the arterial wall. The sections were analyzed by H&E and elastic stain. Elastic fibers were stained *black*, and collagenous fibers were stained *pink*

Compared with the Con group (Fig. 5a, e), larger spaces between elastic fibers were observed in Lig group (Fig. 5f); furthermore, fractures and disordered arrangement in fibers was also observed in Lig group. However, after removal of the causal ligatures and SRP, the inhomogeneous arrangement in fiber was less obvious in Lig-N (Fig. 5c, g) and Lig-SRP group (Fig. 5d, h), demonstrating less elastic fiber fragmentation.

### Aortic inflammatory response

**Fig. 6:**
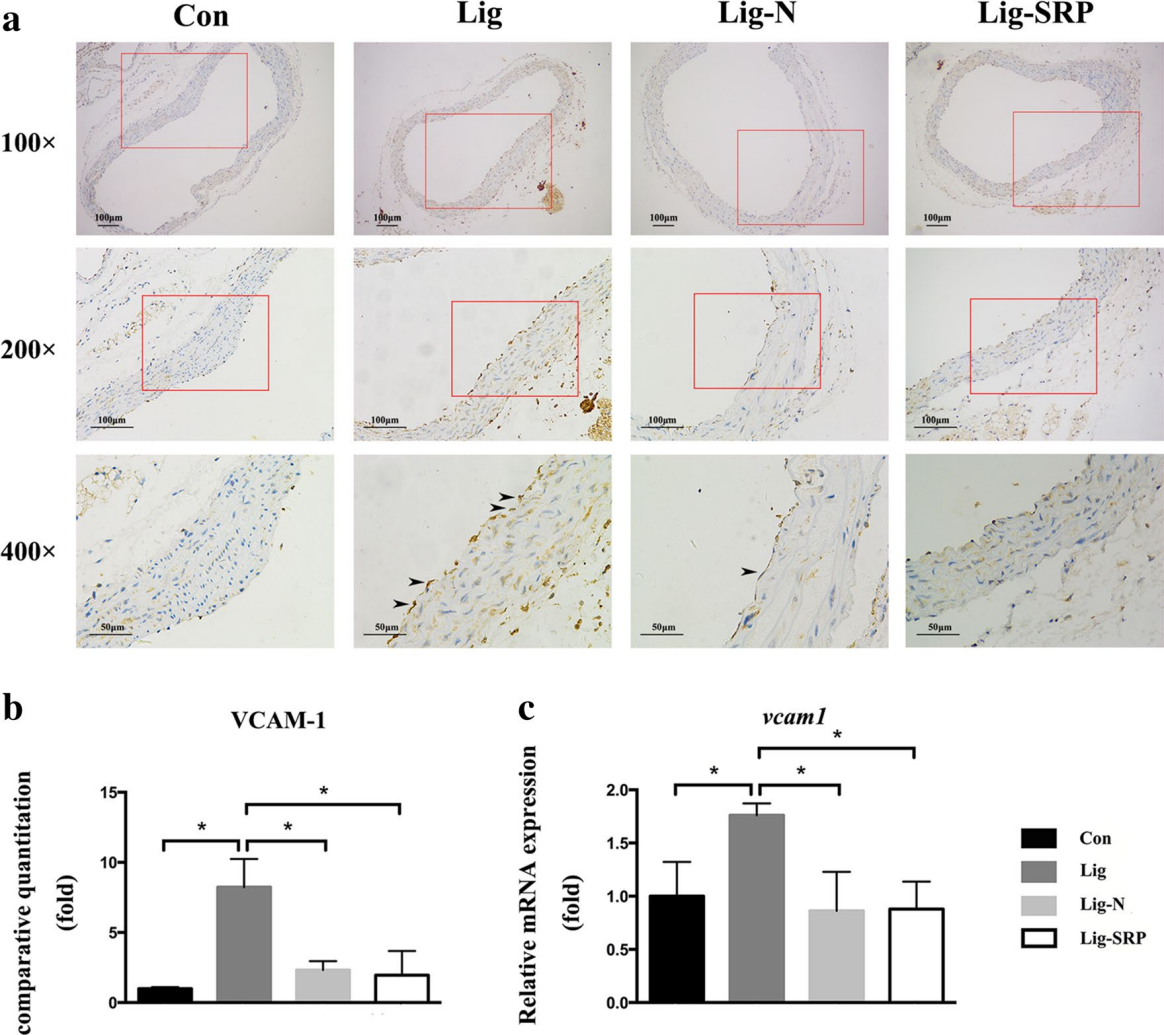
Expression of vascular cell adhesion molecule-1 (VCAM-1) in aorta in different experimental groups. **a** Photomicrographs showing VCAM-1 in the Con group, the Lig group, the Lig-N group and the Lig-SRP group. ×100, *scale bars* 100 μm. ×200, *scale bars* 100 μm. ×400, *scale bars* 50 μm. *Arrowheads* immuno-reactive cells. **b** Graph showing the VCAM-1 positive cell counts by fold in each group. *p < 0.05. **c** Relative gene expression levels of VCAM-1 in aorta. *p < 0.05

**Fig. 7:**
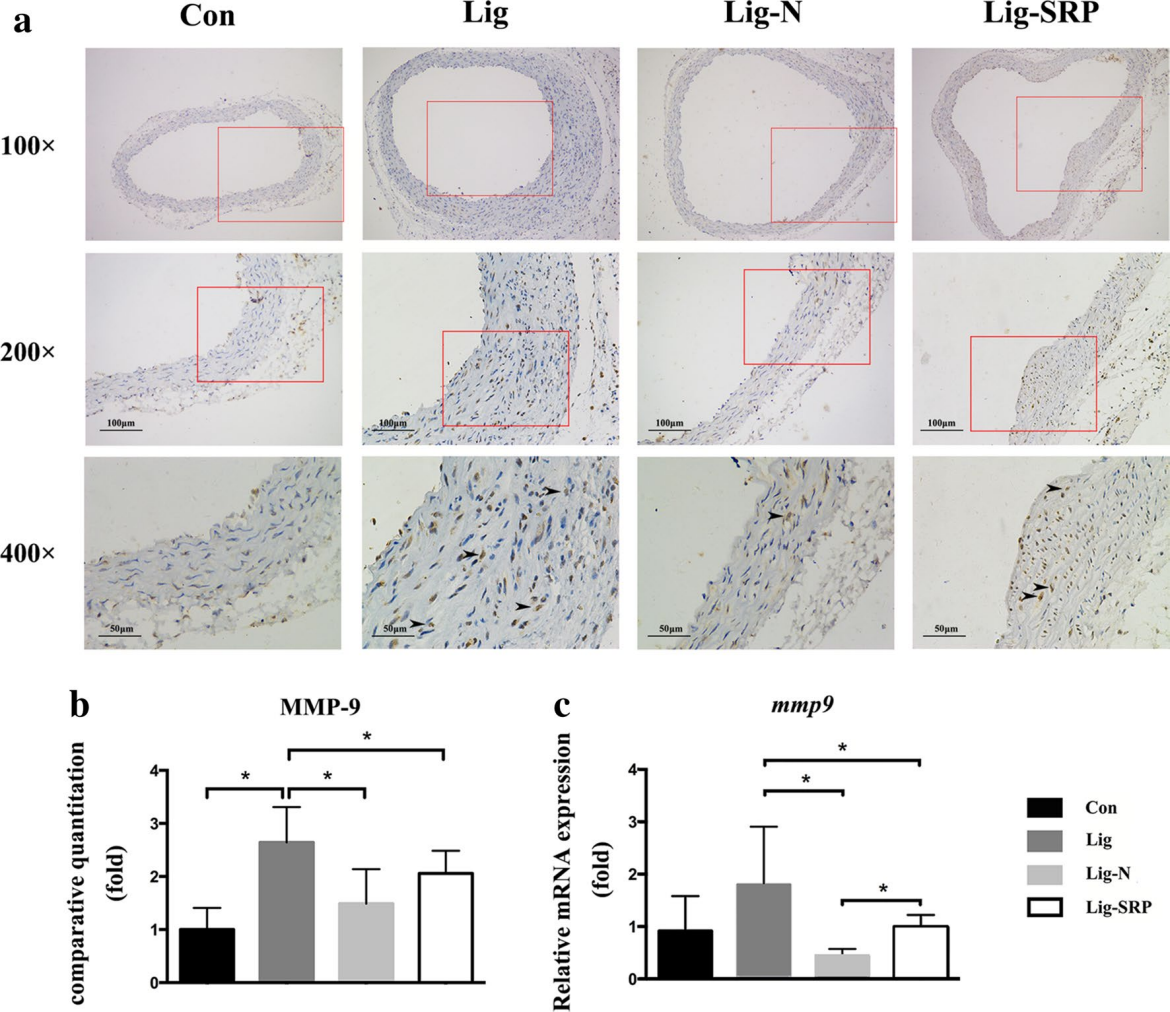
Expression of metallopeptidase-9 (MMP-9) in aorta in different experimental groups. **a** Photomicrographs showing MMP-9 in the Con group, the Lig group, the Lig-N group and the Lig-SRP group. ×100, *scale bars* 100 μm. ×200, *scale bars* 100 μm. ×400, *scale bars* 50 μm. *Arrowheads* immuno-reactive cells. **b** Graph showing the MMP-9 positive cell counts by fold in each group. *p < 0.05. **c** Relative gene expression levels of MMP-9 in aorta. *p < 0.05

**Fig. 8:**
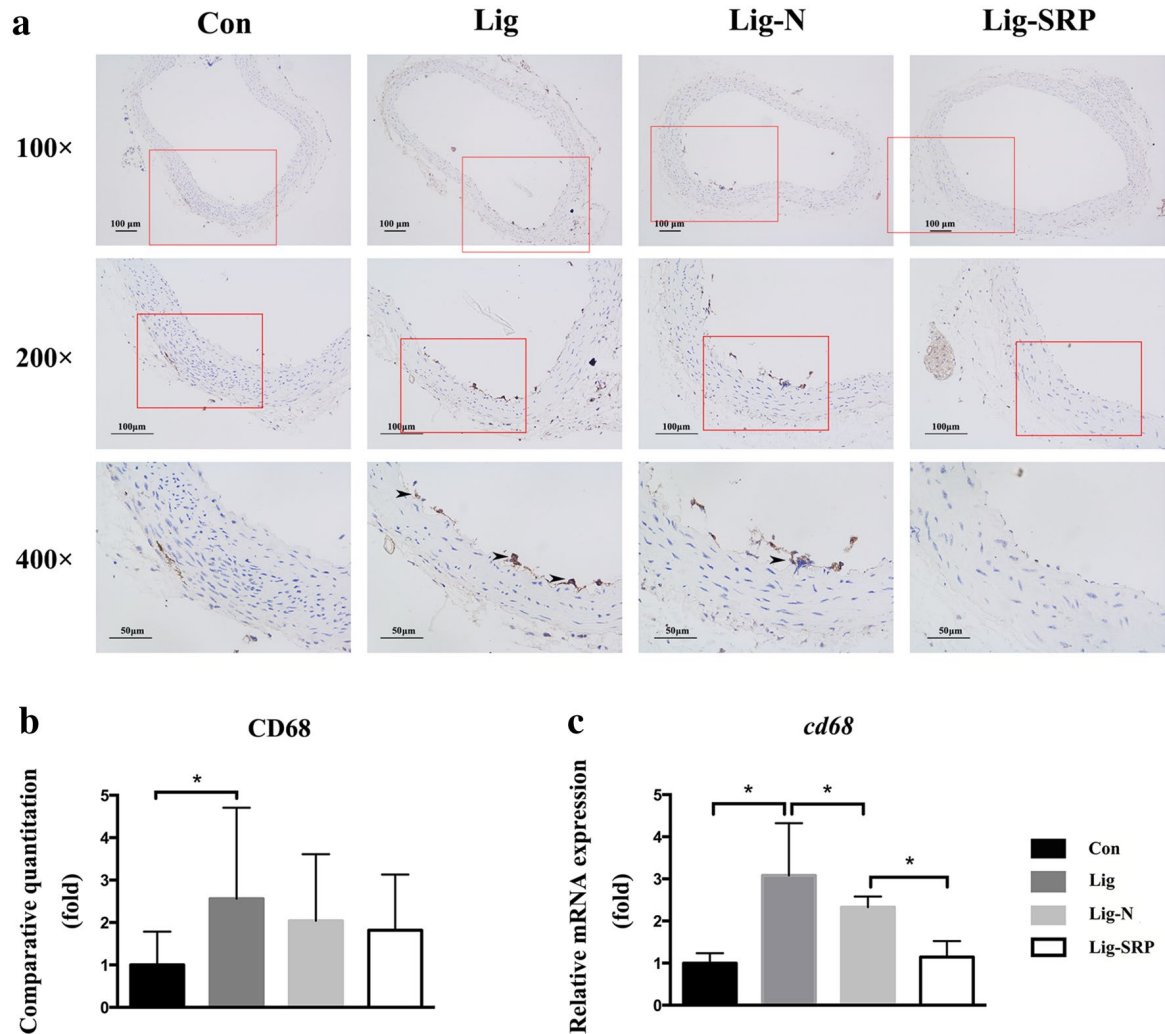
Expression of CD68 in aorta in different experimental groups. **a** Photomicrographs showing CD68 in the Con group, the Lig group, the Lig-N group and the Lig-SRP group. ×100, *scale bars* 100 μm. ×200, *scale bars* 100 μm. ×400, *scale bars* 50 μm. *Arrowheads* immuno-reactive cells. **b** Graph showing the CD68 positive cell counts by fold in each group. *p < 0.05. **c** Relative gene expression levels of CD68 in aorta. *p < 0.05

We further investigated whether the removal of ligatures and SRP would lead to improved vascular response. According to the results of immunohistochemical analysis (Fig. 6a, b) and PCR (Fig. 6c), VCAM-1 expression levels increased significantly in the Lig group, when compared with the Con group (p < 0.05). Removal of ligatures significantly reduced VCAM-1 expression in the vascular wall (p < 0.05). MMP-9 expression levels increased significantly in the Lig group, when compared with the Con group (Fig. 7b, c) (p < 0.05). Removing ligatures significantly reduced mRNA expression level of MMP-9 in the vascular wall; however, levels of MMP-9 was significantly higher in the Lig-SRP group than the Lig-N group (p < 0.05) (Fig. 7c). Compared with the Con group, levels of CD68 were significantly up-regulated after ligature placement (Fig. 8) (p < 0.01), and dropped to the Con group level after ligature removal and SRP. When compared with the Lig-N group, relative mRNA expression level of CD68 was significantly decreased in the Lig-SRP group (p < 0.01).

## Discussion

This is the first animal study to investigate whether periodontal treatment would improve atherosclerotic status in the arterial wall. We demonstrate that periodontal treatment including removal of the causal ligatures and SRP plays a protective role in the atherosclerosis process in hyperlipidemic mice with periodontitis.

Ligature-induced-experimental periodontitis mice model has been applied in periodontitis related researches (Matsuda et al. 2015; Lin et al. 2014). Abe and Hajishengallis (2013) observed the accumulation of anaerobic bacteria around ligatures 2 d after the placement of ligature. Our findings agree with the study carried out by Garcia et al. (2015) that with the smallest curette and great attention, SRP can be performed in small animals. In their study, SRP was performed in rats. And we modified their method to apply SRP to mice. Furthermore, we observed less pro-inflammatory cytokine IL-6 and more anti-inflammatory IL-10 after SRP, which agreed with the clinical studies that showed periodontal treatment enhanced IL-10 level and reduced IL-6 level in blood (Passoja et al. 2010; Correa et al. 2010; Acharya et al. 2015). As an anti-inflammatory cytokine in nature, higher levels of IL-10 after SRP may contribute to the decreased inflammation in vascular wall. Therefore, our present study demonstrates that SRP may help to improve the inflammatory response in patients with atherosclerosis.

Early, preclinical atherosclerosis changes in the vasculature may be associated with TG levels (Al-Aubaidy and Jelinek 2014; Miller et al. 2011). It is reported that severe periodontitis has been associated with a robust increase in plasma TG (Maekawa et al. 2011; D’Aiuto et al. 2006), which is agreed by the present study. NSPT improved plasma lipid, which might be another mechanism by which NSPT decreased inflammatory response in the arterial wall (Moraes et al. 2015; Cho 2002; Lemaitre et al. 2009).

Meanwhile, we analyzed indicators in aorta, in order to figure out what effect periodontitis and NSPT could have on the development of atherosclerosis. Elasticity, provided by the abundant pool of elastic fibers, is an important characteristic for many vital organs such as the great arteries, lungs, and skin. To maintain their physiological function, these organs need to undergo repeated cycles of extension and contraction (Horiguchi et al. 2009). In the present study, we observed increased depletion of elastic fibers in mice with periodontitis, leading to loss of elasticity in the arteries, and we further demonstrated NSPT has a protective role in elastic fiber fragmentation, thereby decreased the incidence of emergent cardiovascular incidents.

We further investigated the mechanism by which NSPT decreased arterial elastic fiber fragmentation. In essence, atherosclerosis is an inflammatory disease. MMPs play an important role in degrading the extracellular matrix and, when unregulated, can lead to aortic wall destruction in atherosclerosis (Albini et al. 2014). In the present study, increased MMP-9 level in the arterial wall was observed in the mice after ligature placement; however, SRP did not help MMP-9 reduction. The balance between MMPs and tissue inhibitors of matrix metalloproteinases (TIMPs) finely orchestrates the degradation and formation of extracellular matrix in the arterial wall during atherosclerosis progression (Liu et al. 2009). Further study on TIMPs may help to unveil the full role of SRP on arterial wall. In addition, bacteremia following SRP is one common phenomenon in periodontal therapy (Zhang et al. 2013; Graziani et al. 2015) and this may pose a problem due to the small size of the mice. Our present study stressed the complexity of the relationship between periodontal diseases and atherosclerosis.

VCAM-1 up-regulation has shown to be a marker of inflammatory response in the arterial wall (Miyajima et al. 2014; Yang et al. 2015). The induction of VCAM-1 is an example of inducible NF-κB-dependent gene expression during the aortic inflammation, since the promoter of VCAM-1 contains two consensus NF-κB sites that are required for cytokine-induced expression (Iiyama et al. 1999). Various stimulatory factors, including increased plasma lipid contents, hypertension, and pathogens, can lead to increased VCAM-1 expression in the endothelial cells (Gimbrone 1995),which attracts monocytes to adhere to the endothelium and further migrate to the vascular wall. In this study, elevated expression level of VCAM-1 by periodontitis were detected in the Lig group, suggesting that periodontitis promotes the development of atherosclerosis by exacerbating aortic inflammation. Following the reduced VCAM-1 expression in the vascular wall, less macrophages/monocytes infiltration was observed in mice received NSPT, as shown by the reduced CD68 in the vascular wall.

The major possible mechanism by which periodontitis affects vascular status is the dissemination of periodontal pathogens and their byproducts into the circulation system, thereby eliciting inflammatory responses in the arterial wall (Monteiro et al. 2009). The DNA of periodontal pathogen such as *P. gingivalis* has been detected in human cardiovascular specimens (Nakano et al. 2008). In addition, accelerated atherosclerosis progress has been found in apoE^**−/−**^ mice by oral or intravenous inoculation of *P. gingivalis or Fusobacterium nulceatum* (Lalla et al. 2003; Li et al. 2002; Velsko et al. 2015). Furthermore, a ligature-induced periodontitis rat model, increased lipid peroxidation and IL-6 expression was observed in aortic wall (Ma et al. 2010). In the present study, decreased periodontal destruction after NSPT was observed, therefore, less periodontal pathogens and their byproducts was released into the blood stream, leading to less inflammatory response in the arterial wall.

In the present study, we focused on the short-term effects of NSPT on inflammatory response in the aortic wall. Due to the chronic progress of atherosclerosis, Oil Red O staining of the aortic tree for en face observation of atherosclerotic plaque may further help to understand the long-term effect of NSPT on the progression of atherosclerosis.

## Conclusion

In conclusion, synchronization of improved lipid profile, decreased systemic cytokines, and mitigated inflammation in periodontal tissues contributes to the decreased inflammatory response in the arterial wall of NSPT-treated mice, including elastic fiber fragmentation, extracellular matrix degradation and endothelial dysfunction.

## Methods

### Animals

Thirty-two 9-week-old male apoE^−/−^ mice (Peking University Health Science Center, Peking, China) were included in the study. All mice were housed in individual cages under controlled temperatures (24 ± 1.0 °C) in a 12 h light/dark cycle and were provided free access to standard laboratory mice chow and aseptic water. All experiments were performed in accordance with the policies and guidelines for institutional animal care of Nanjing University. ApoE^−/−^ mice are predisposed to developing hyperlipidemia, even when fed a standard mouse chow diet (Lei et al. 2013; Pan et al. 2014).

### Experimental protocols

**Fig. 9:**
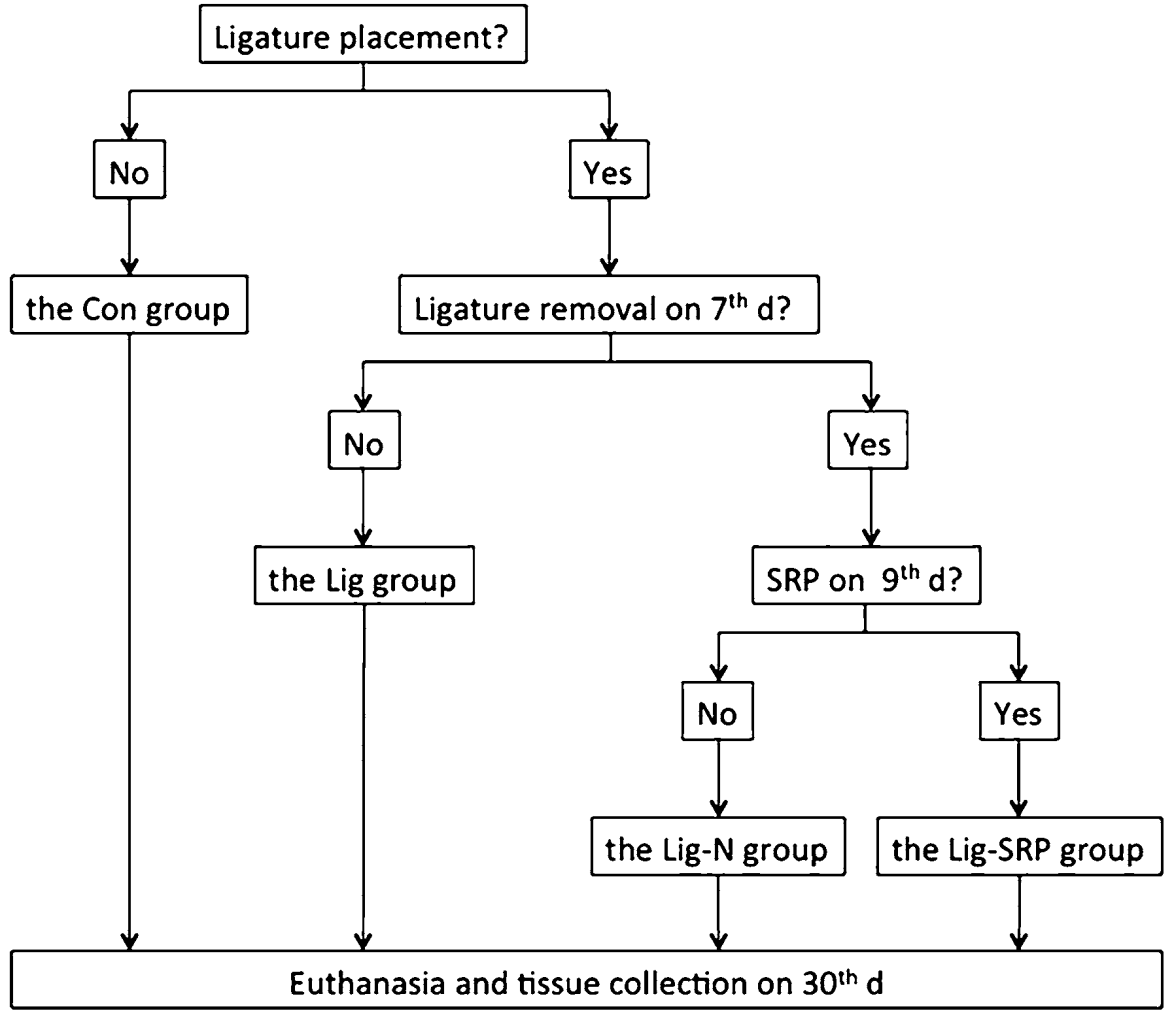
Flowchart showing the design of the study

When the animals were 10 weeks old, they were randomly divided into four groups as follows: (1) Con group: no treatment, blank control group; (2) Lig group: ligature-induced-periodontitis group; (3) Lig-N group: ligatures were removed on the 7th day; (4) Lig-SRP group: ligatures were removed on the 7th day and SRP were performed on the 9th day. All the animals were sacrificed on the 30th day (Fig. 9).

### Induction of periodontitis

For all procedures, the mice were anesthetized with pentobarbital sodium (70 mg/kg; Nanjing SenBeiJia Biological Technology Co., Ltd., Nanjing and atropine sulfate monohydrate (6 mg/kg; Tianjin KingYork amino acid Co., Ltd., Tianjin), which were administered via peritoneal injection. Periodontitis was induced in the mice by tying a 5-0 suture silk ligature (Jinghuan Medical Appliance, Yang Zhou, China) around the cervical area of bilateral 2nd maxillary molars on both sides. The ligatures were checked once other day to confirm the development of periodontal bone loss according to experimental design.

### Scaling and root planing

The ligatures were removed on the 7th day. A modified Garcia’s method (Garcia et al. 2015) was used in the treatment of periodontitis. The 2nd maxillary molars were subjected to SRP with manual 12 mini-five curettes (Hu-Friedy Co. Inc., Chicago, IL, USA) through 10 distal–mesial traction movements in the buccal and palate aspects and with a probe through cervico-occlusal traction movements in the furcation and interproximal areas. The probe was worn dull previously to minimize the damage to the root and soft tissues. All the procedures were performed by the same experienced operator.

### Determination of bacterial accumulation

In order to detect the plaque accumulation on the tooth surface, MIRA 2 TON (Hager & Werken GmbH & Co. KG, German) was used according to the manufacturer’s protocol. The solution was applied to all exposed tooth surfaces, followed by rinsing with deionized water. Older plaque is revealed in blue, newer plaque in pink. The ligatures in the Lig groups were recovered and gently washed with sterile PBS in order to remove food residue and other debris. Then the sutures were cut into a length of 4 mm. Plaques on the tooth surface and in the gingival crevicular (or periodontal pocket) were sampled with a sterilized paper point. In the Lig group, both the sutures and paper points were placed in Eppendorf tubes with 500 μl PBS. In the other groups, only paper points were collected. The tubes were vortexed for 5 min at 3000 rpm to extract the bacteria. Serial dilutions of the bacterial suspensions were plated onto blood agar plates and colony forming units were enumerated following anaerobic growth at 37 °C for 3 days.

### Animal sacrifice and analysis

The mice were euthanized by an overdose of isoflurane. The maxillary jaws were hemisected and the right one-half of the block samples (N = 6–8) were fixed in 4.0 % paraformaldehyde and submitted to routine microcomputed tomography (micro-CT) for alveolar-bone-loss (ABL) evaluation and histological analysis. The arcus aortae were dissected, fixed overnight at 4.0 °C with 4.0 % paraformaldehyde in phosphate buffered saline (PBS), dehydrated, and embedded in paraffin blocks. The descending aortas and the other half of maxillary jaws were dissected and snap-frozen in liquid nitrogen until processing for real-time polymerase chain reaction (RT-PCR) analysis. Blood was collected in citrate anticoagulation tubes and the plasma was separated by centrifugation at 3000 rpm for 5 min.

### Microcomputed tomography analysis

To observe the morphological changes in the alveolar bone, maxillae were scanned by micro-CT (Bruker micro-CT, Kontich, Belgium). The computed tomography was set according to slice thickness (18 µm), voltage (50 kV), and electrical current (455 µA). Three-dimensional images were made using the Bruker micro-CT version 1.1 (Bruker micro-CT, Kontich, Belgium).

### Analysis of bone height

The distance from the cement-enamel junction to the alveolar bone crest of the 2nd molars was measured at the four sites, namely mesial-bifurcation, buccal-bifurcation, distal-bifurcation, palatal-groove. Bone heights were analyzed using the measure function of the Dataviewer CT (Bruker micro-CT, Kontich, Belgium). And the mean alveolar bone loss was calculated respectively at the four sites.

### Plasma lipid level

The plasma TC, TG, LDL-c and HDL-c concentration was determined by an enzymatic method using the protocols provided by an automatic biochemical analyzer (Mindray Medical International Limited, Shenzhen, China).

### ELISA

Levels of interleukin (IL)-6, IL-10, CRP and soluble Triggering receptor expressed on myeloid cells 1 (sTrem-1) in plasma were assayed using ELISA kits (R&D Systems, Minneapolis, MN, USA) according to the manufacturer’s recommendations. Each was determined according to its optical density, measured using a microplate spectrophotometer at 450 nm.

### Aorta elastic staining

To investigate the changes in the elastic fibers of arterial wall, elastic stain was performed as described by Albini et al. (2014). After dewaxing and rehydration, sections were incubated with Verhoeff’s dyestuff (Goodbio Biotechnology Company, Hubei Province, China), followed by 2 % ferric trichloride for 10–20 s. After washing in PBS and redyeing with Verhoeff’s dyestuff for 10–15 s, all sections were washed in ethyl alcohol and mounted under coverlips. The sections were evaluated using a microscope with a digital camera.

### Immunohistochemistry

Sections were dewaxed and rehydrated. Then, paraffin sections were heated in EDTA antigen retrieval solution (Cat number: G1203, Goodbio Biotechnology Company, Hubei Province, China) using a pressure cooker. To block endogenous peroxidase activity, sections were then washed with PBS and treated with 3 % hydrogen peroxide for 10 min. They were immersed in one of the following primary antibodies for 12 h at 4 ℃: goat polyclonal anti-VCAM-1 (1:15, R&D Systems, Cat number: AF643, Minneapolis, MN, USA), goat polyclonal anti-MMP-9 (1:15, R&D Systems, Cat number: AF909, Minneapolis, MN, USA) and rabbit polyclonal anti-CD68 (1:100, abcam^®^, Cat number: ab125212, Cambridge, MA, USA). All the antibodies were diluted in sterile PBS. After washing in PBS, the sections were incubated for 15 min at room temperature with HRP-polymer anti-goat (Cat number: GB23204, Goodbio Biotechnology Company, Hubei Province, China) or HRP-polymer anti-rabbit (Cat number: GB23303, Goodbio Biotechnology Company, Hubei Province, China), then washed in PBS. The secondary antibodies were diluted to 1:200 in sterile PBS. All the sections were applied with DAB buffer (Cat number: K5007, Goodbio Biotechnology Company, Hubei Province, China). After washed with PBS, they were counterstained with hematoxylin for 20 s and then rinsed in running water. Finally, the sections were dehydrated in ascending concentrations of alcohol, cleared with xylene and mounted. Controls for the immunostaining procedures were obtained by omission of the primary antibodies and substitution with nonspecific antibodies.

### Immunohistochemical analysis

A trained examiner, who was blinded to the treatment, selected the sections for the immunohistochemical analyses. Each section was measured three times by the same examiner. The sections were evaluated using a microscope with a camera mounted on a computer (1X71, Olympus Co., Tokyo, Japan). Three histologic sections from each mouse were used. Random 3 visual fields at 400× magnification from each section were photographed. The number of immuno-reactive cells was counted in the entire area of each photography.

### Quantitative real-time PCR

Total RNA isolated from left maxillary and aorta was extracted using the TRIzol reagent (Invitrogen, Carlsbad, CA, USA) and reverse transcribed into cDNA using a PrimeScriptTM 1st Strand cDNA Synthesis kit (Takara, Dalian, China) according to the instructions. Primers for CD68, IL-6, IL-10, VCAM-1 and MMP-9 (Table 1) were designed using Oligo Explorer software (1.1.0) and synthesized by Genscript Co. (Nanjing, Jiangsu, China). Real-time RT-PCR analysis was performed by SYBR Green based assays using the Stratagene MxPro-Mx3005P System (Agilent Technologies, Santa Clara, CA, USA). PCR reactions were conducted with 2 μl of diluted cDNA samples, 200 nM of each respective forward and reverse primer in a 20 μl final reaction mixture with Platinum SYBR Green qPCR SuperMix-UDG (Invitrogen). The internal reference gene GAPDH was used as endogenous control. The PCR reactions for each gene were initiated by activation at 95 °C for 2 min, followed by 40 PCR cycles of denaturation at 95 °C for 15 s, annealing and extension at 60 °C for 30 s. The results were analyzed using MxPro 4.10 software.

**Table 1:** Primers used for real-time RT-PCR

Genes	Oligos	Primers 5′–3′	Expected amplicon size (bp)	UniGene numbers
GAPDH	F-primer	GTTGTCTCCTGCGACTTCA	293	Mm.304088
	R-primer	GCCCCTCCTGTTATTATGG		
IL-6	F-primer	TTCCATCCAGTTGCCTTCTTG	134	Mm.147620
	R-primer	GGGAGTGGTATCCTCTGTGAAGTC		
IL-10	F-primer	GCCCTTTGCTATGGTGTCCT	106	Mm.124092
	F-primer	GCCCTTTGCTATGGTGTCCT		
VCAM-1	F-primer	CTGGGGCAGGAAGTTAGATA	270	Mm.76649
	R-primer	CAAGAAAAAGAAGGGGAGTCA		
CD68	F-primer	GCTGTTCACCTTGACCTGCTC	192	Mm.15819
	R-primer	GGTTGATTGTCGTCTGCGG		
MMP-9	F-primer	CCGAGCTATCCACTCATCAAAC	263	Mm.4406
	R-primer	CTGAACCATAACGCACAGACC		

### Statistical analysis

Data are expressed as the mean ± SD. The differences between the two groups were assessed using the unpaired two-tailed Student’s *t* test, unless otherwise stated. Data sets involving more than two groups were assessed by one-way analysis of variance followed by the Bonferroni correction for multiple comparisons. The differences were considered to be significant if p < 0.05.

## Abbreviations

NSPT: nonsurgical periodontal treatment
apoE^−/−^: apolipoprotein E^−/−^
IL-6: interleukin-6
VCAM-1: cell adhesion molecule-1
MMP-9: matrix metallopeptidase-9
TNF-α: tumor necrosis factor-α
CRP: C-reaction protein
SRP: scaling and root planing
micro-CT: microcomputed tomography
ABL: alveolar-bone-loss
PBS: phosphate buffered saline
RT-PCR: real-time polymerase chain reaction
sTrem-1: triggering receptor expressed on myeloid cells 1
H&E: hematoxylin and eosin
TC: total cholesterol
TG: triglycerides
LDL-c: low-density lipoprotein cholesterol
NF-κB: nuclear factor-kappa B p65
